# Varying Relationship Between Vascular Plant Leaf Area and Leaf Biomass Along an Elevational Gradient on the Eastern Qinghai-Tibet Plateau

**DOI:** 10.3389/fpls.2022.824461

**Published:** 2022-04-12

**Authors:** Ketong Yang, Guopeng Chen, Junren Xian, Weiwei Chen

**Affiliations:** ^1^College of Forestry, Gansu Agricultural University, Lanzhou, China; ^2^College of Environmental Sciences, Sichuan Agricultural University, Chengdu, China

**Keywords:** scaling relationship, leaf trait, elevational gradient, arid valley, alpine plant

## Abstract

The altitudinal gradient is one of the driving factors leading to leaf trait variation. It is crucial to understand the response and adaptation strategies of plants to explore the variation of leaf traits and their scaling relationship along the altitudinal gradient. We measured six main leaf traits of 257 woody species at 26 altitudes ranging from 1,050 to 3,500 m within the eastern Qinghai-Tibet Plateau and analyzed the scaling relationships among leaf fresh weight, leaf dry weight, and leaf area. The results showed that leaf dry weight increased significantly with elevation, while leaf fresh weight and leaf area showed a unimodal change. Leaf dry weight and fresh weight showed an allometric relationship, and leaf fresh weight increased faster than leaf dry weight. The scaling exponent of leaf area and leaf fresh weight (or dry weight) was significantly greater than 1, indicating that there have increasing returns for pooled data. For α and normalization constants (β), only β of leaf area vs. leaf fresh weight (or dry weight) had significantly increased with altitude. All three paired traits had positive linear relationships between α and β. Our findings suggest that plants adapt to altitudinal gradient by changing leaf area and biomass investment and coordinating scaling relationships among traits. But leaf traits variation had a minor effect on scaling exponent.

## Introduction

Leaves are crucial for plant metabolic performance, have significant functions in biogeochemical cycles (Cui et al., 2020; Cubino et al., 2021), and influence global climate change (Niinemets, 2001; Li et al., 2008; Cubino et al., 2021). Previous studies have confirmed that leaf size spans six orders of magnitude (Milla and Reich, 2007; Wright et al., 2017), and this variation in leaf size is the basis for maintaining biodiversity (Mi et al., 2021). For leaves, abiotic factors (e.g., altitude) are one of the driving forces of variation (Chen et al., 2021; Jiang et al., 2021; Ren et al., 2021). The scaling relationship between leaf traits caused by leaf size may affect leaf biomass and element allocation, and this relationship has also been proven to be one of the strategies for species to acquire resources, and even affect species coexistence and community construction (Li et al., 2008; Ren et al., 2021). Although variations in leaf and scaling relationships between different climatic regions and life forms have been widely documented (Niklas et al., 2007; Li et al., 2008), how they vary along elevation gradient remains unclear.

Plant leaves have abundant phenotypic variation. Milla and Reich’s (2011) study showed that leaves gradually became thicker and water content decreased with elevation, and Guo et al.’s (2018) study showed that the leaves gradually became smaller with altitude. These variations are thought to be better adapted to the environment, with smaller, thicker leaves that can endure mechanical damage from intense radiation, freezing, and wind (Xiang et al., 2009; Pan et al., 2013). However, at present, many studies only focus on the changes of leaf traits along the elevation gradient, and such static changes only represent the leaves’ current situation, while the dynamic changes among traits, such as allometric relationship (or scaling relationship), are ignored. Another very important aspect is that although Sun et al. (2017) and Guo et al. (2018) have done some work on allometric variation along elevation gradients, a single species (bamboo) is not a good representative plant for the whole community. Therefore, it is urgent to explore whether leaves allometric growth at the community level is affected by altitude.

Elevational gradient represents a combination of various changing environmental factors, namely, colder climate, decreasing soil depth, and less fertile soil, but also reduced human disturbance with increasing altitude (Kühn et al., 2021). Meanwhile, the elevation gradient is known as a “natural platform” for studying plant variation (Thakur et al., 2019). With increasing altitude, most functional traits, namely, leaf area (LA), leaf dry weight (LDW), leaf fresh weight (LFW), and water content, decreased significantly (Guo et al., 2018). Because most traits are correlated, these will further lead to variation in the scaling relationship. For instance, Pan et al. (2013) found that the scaling exponent between LA with LDW gradually increased from 0.859 to 1.258 along the elevational gradient for 121 vascular species ranging from 414 to 1,462 m on Mt. Tianmu, and they attributed the reason to environmental variations that cause different leaf biomass allocation. However, Thakur et al. (2019) showed that the scaling exponent of LA and LDW decreased significantly from 1.08 to 0.85 with increasing elevation with the altitude from 3,350 to 5,150 m in the western Himalaya. Therefore, we attempted to further summarize the general relationship between LDW–LFW, LA–LFW, and LA–LDW through a larger elevation scale, and test the relationship between leaf size and scaling exponent to systematically elucidate the variation mechanism of leaf traits and their internal relationships with altitudes. These will help to expand our understanding of plant light capture cost mechanisms and their response and adaptation to elevation gradient.

The eastern region of the Qinghai-Tibet Plateau has diverse native flora and is significant for protecting biodiversity and ecosystem balance (Chen et al., 2021; Liu et al., 2021). Some vegetation of this region has been severely disturbed in the past. After decades of restoration and conservation, most of the vegetation is recovering (Yan et al., 2013; Chen et al., 2021). Therefore, the region is one of the most powerful “natural laboratories” for studying the elevation responses of the plant (Thakur et al., 2019). To explore changes of scaling exponents and normalization constants along the elevation gradient, we set 26 plots in different altitudes along 1,050–3,500 m located at the eastern Qinghai-Tibet Plateau. In this research, we measured leaf traits—i.e., LA, LFW, LDW, specific leaf area (SLA), and leaf dry matter content (LDMC)—of 257 woody plant species with the following objectives: (1) How do the leaf traits change along the altitudinal gradient? (2) Whether elevation gradient will affect the variation of scaling exponent and normalization constant?

## Materials and Methods

### Study Sites

The study sites located in Gongbahe of Bailong River (GBR) in Zhouqu County, Southern Gansu Province, P.R. China (103° 57′ 05″–104° 42′ 05″ E, 33° 14′ 32″–33° 53′ 52″ N, 998–3,600 m a.s.l.), which is the transition zone of temperate monsoon, subtropical monsoon, and plateau montane climate zones, and at the boundary between semihumid and semiarid regions (Chen et al., 2021). According to the last 30 years of climatic data, the mean annual rainfall, evaporation of GBR, relative humidity, and mean annual temperature are 951 mm, 918 mm, 82%, and 4.3°C, respectively. The mean temperature of the coldest month (January) is −13.3°C; the mean temperature of the hottest month (July) is 20.8°C; and the annual frost-free period is about 96.7 days. The annual sunshine duration is 1,398.4 h, and the sunshine percentage is 32.0%. The old-growth vegetation of GBR had been logged several decades ago and the regrowth of restored vegetation is well underway (Yang et al., 2021).

During previous *in situ* surveys, we found that vegetation gradually changed from arid valley dwarf xerophytic shrubs, deciduous broad-leaved forests, to the evergreen coniferous forest and evergreen broad-leaved shrubs from 1,050 to 3,500 m (Chen et al., 2021; Yang et al., 2021). We set up a transect along an elevational gradient and surveyed 26 plots of 20 m × 20 m at different altitudes (Appendix 1).

### Sampling and Measurement of Leaves

We identified a total of 257 woody plant species (some plants were found in multiple plots), belonging to 55 families and 115 genera (Appendix 2) according to *Flora of China*,^1^ selected three healthy branches of each woody species, and collected five leaves from the middle-upper canopy of each plant at 10:00–14:00. Then, put the leaves into plastic self-sealing bags in a portable incubator with ice bags (to prevent blades from deforming and losing water), and then brought them to the forest research station to measure.

Each leaf was scanned, and images were saved as bitmap images at a 480-dpi resolution using a scanner (EPSON V39, Indonesia). Image J software (version 1.48)^2^ was used to obtain a leaf profile in a black and white image. Then, the length, width, and area of the blade were measured by the Image J. We then measured LFW and dried the leaves in a ventilated oven at 105°C for 15 min and turned to 75°C until achieving a constant dry weight (i.e., LDW) (Huang et al., 2019a; Guo et al., 2021; Jiang et al., 2021). LFW and LDW were both measured using an electronic balance (0.0001 g, Zhuojing Experimental Equipment Co. Ltd., BMS, Shanghai, China).

### Data Analysis

**FIGURE 1:**
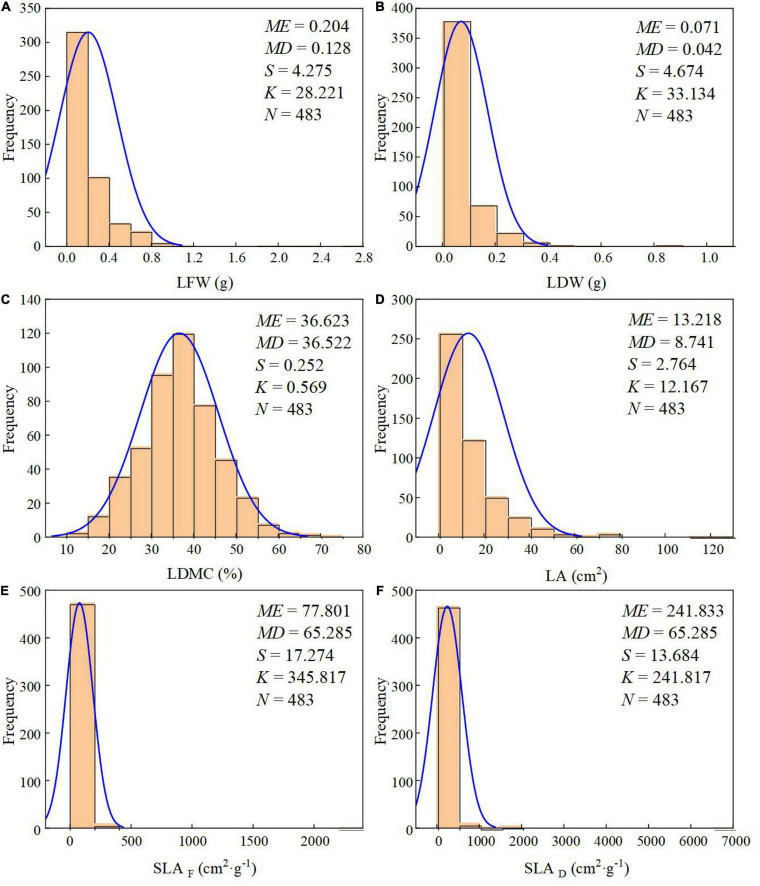
Frequency distribution histogram and normality test of leaf traits. **(A)** LFW; **(B)** LDW; **(C)** LDMC; **(D)** LA; **(E)** SLA_*F*_-based fresh weight; and **(F)** SLA_*D*_-based dry weight. ME, mean value; MD, median value; S, skewness; K, kurtosis; N, total number.

The arithmetic mean value is often used to represent the average of a series of measurements. But when data are not normally distributed, the median may be more representative. Thus, we tested the normality of the dataset, and five out of six traits showed non-normal distribution (Figure 1). So, we first calculated the mean for every individual leaf trait as a species traits value, then, for each altitude (plot), we calculated each plot species median traits value as a community traits value. Some previous studies found that LFW is better for describing the scaling relationship between leaf biomass and LA (Huang et al., 2019b; Shi et al., 2020). So, we calculated SLA by LFW and LDW, which were used by SLA_*F*_ and SLA_*D*_, respectively.

We established scaling relationships between traits based on species at each altitude (plot). The relationships of LDW–LFW, LA–LFW, and LA–LDW can be described as *y* = β *x ^α^*, linearized under the form log (*y*) = log (β) + α log (*x*). The values of α determine whether the relationship is isometric (α = 1.0) or allometric (α > 1.0 or α < 1.0). The term β is the *y*-intercept of the relationship (Xiang et al., 2009; Pan et al., 2013). Its value does not determine the form of the relationship and, if two lines of the same slope are compared, the difference between their respective values of β indicates the difference independent of parameters. The 95% CIs of α and β were calculated using the SMATR Version 2.0 (Falster et al., 2006). For the three paired traits, we compared its α with 1.0 to test the difference. If the slope is not significantly different from 1.0, the relationship between the two indexes represents roughly isometric growth; and if the slope is greater or less than 1.0, the relationship between the two indexes is allometric growth (Xiang et al., 2009; Sun et al., 2017). We used the coefficient of determination (*R*^2^) to determine the goodness of fit. The images describing scaling exponents and normalization constants were analyzed by the *mgcv* (2011) package and of R 4.0.5 software (R Core Team, 2021).

## Results

### Leaf Traits Variation Along the Altitudinal Gradient

**FIGURE 2:**
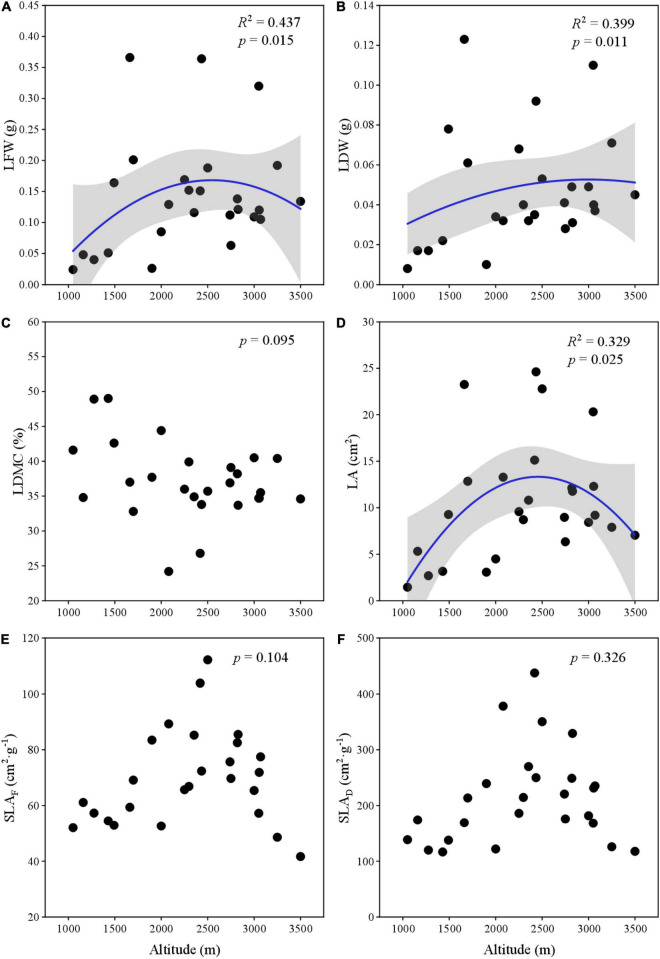
The variation of leaf traits along elevation gradient. **(A)** LFW; **(B)** LDW; **(C)** LDMC; **(D)** LA; **(E)** SLA_*F*_-based fresh weight; and **(F)** SLA_*D*_-based dry weight.

Leaf fresh weight (LFW) and LA showed significant unimodal variation across the elevational gradient (Figures 2A,D) and ranged from 0.024 g and 1.456 cm^2^ to 0.366 g and 24.619 cm^2^, respectively. LDW gradually increased and then decreased (*p* < 0.05) at 3,200 m (Figure 2B). LDMC ranged from 24.21 to 48.96% and had no obvious relationship with altitude (Figure 2C). SLA_*F*_ and SLA_*D*_ ranged from 41.686 and 116.582 cm^2⋅^g^–1^ to 112.179 and 437.291 cm^2⋅^g^–1^, respectively (Figures 2E,F). They neither showed a significant relationship with elevations, but had a maximum unimodal at 2,500 m.

### Scaling Relationship Between Leaf Traits for Pooled Data

**FIGURE 3:**
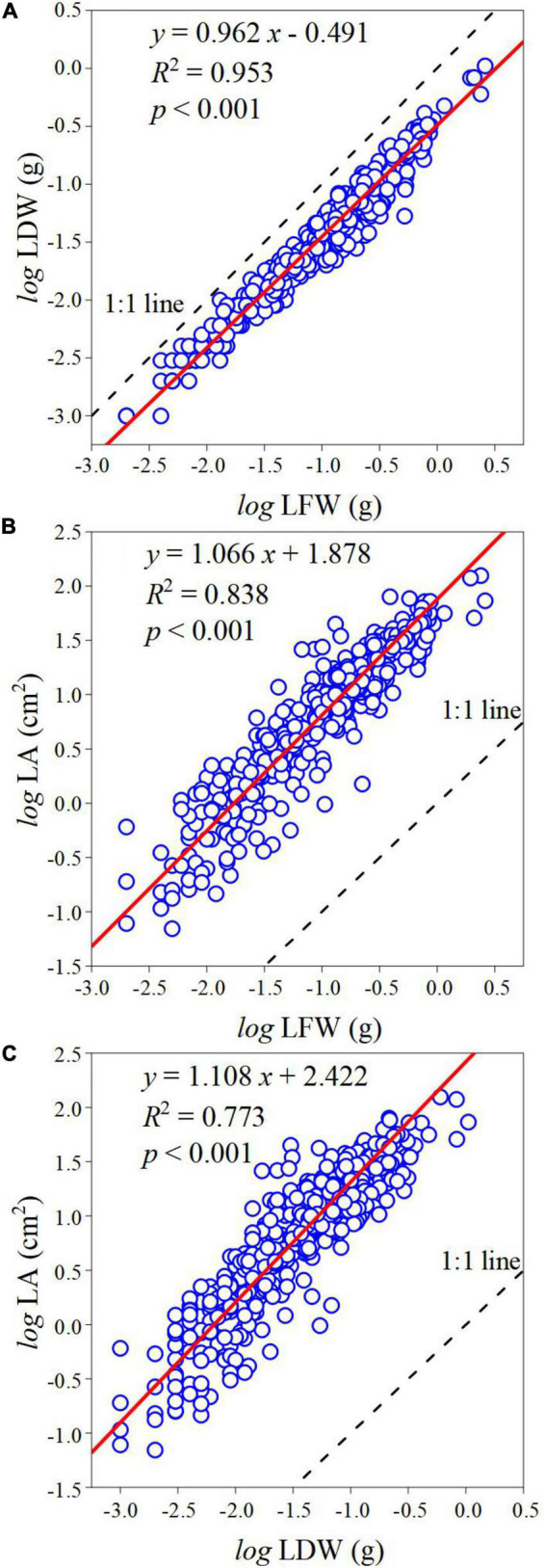
The scaling relationship between leaf traits. **(A)** The scaling relationship between LFW and LDW. **(B)** The scaling relationship between LFW and LA. **(C)** The scaling relationship between LDW and LA. The blue circle represents observed values, the red line represents the SMA regression line, and the black-dotted line represents the 1:1 line; *R*^2^ is the coefficient of determination that is used to measure the goodness of fit; *p* represents the significant level of goodness of fit at 0.05 level.

The log-transformed relationships of LDW–LFW, LA–LFW, and LA–LDW exhibited strong linear (Figure 3). The scaling exponent of LDW–LFW was 0.962 (95% CI, 0.951–0.973) (Figure 3A), which was significantly less than 1.0 (*p* < 0.001). The scaling exponents of LA–LFW and LA–LDW were 1.066 (95% CI, 1.044–1.088) and 1.108 (95% CI, 1.082–1.136) (Figures 3B,C), both significantly greater than 1.0 (*p* < 0.001).

### Scaling Exponent and Normalization Constant Variation Along the Altitudes

**FIGURE 4:**
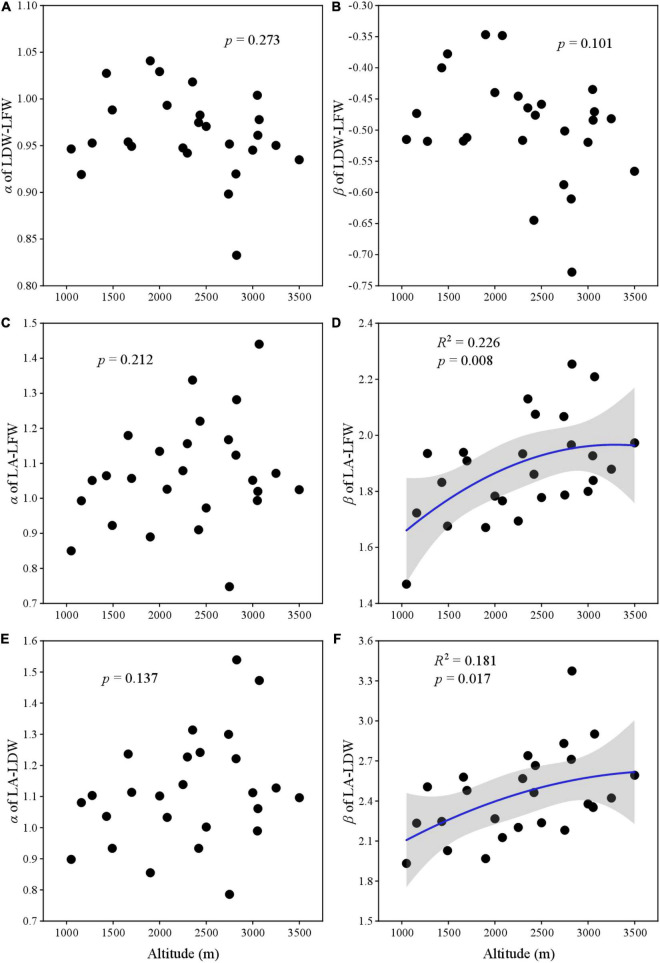
The relationship between scaling exponent and normalization constant with elevation gradient. **(A)** Scaling exponent of LDW and LFW; **(B)** normalization constant of LDW and LFW; **(C)** scaling exponent of LA and LFW; **(D)** normalization constant of LA and LFW; **(E)** scaling exponent of LA and LDW; and **(F)** normalization constant LA and LDW.

All α of LDW–LFW, LA–LFW, and LA–LDW showed no relationship with altitudinal gradient (Figures 4A,C,E). For LA–LFW and LA–LDW, the β first increased and then slowly converged with altitude (Figures 4B,D,F).

### The Relationship Between Leaf Traits and Scaling Parameters

**FIGURE 5:**
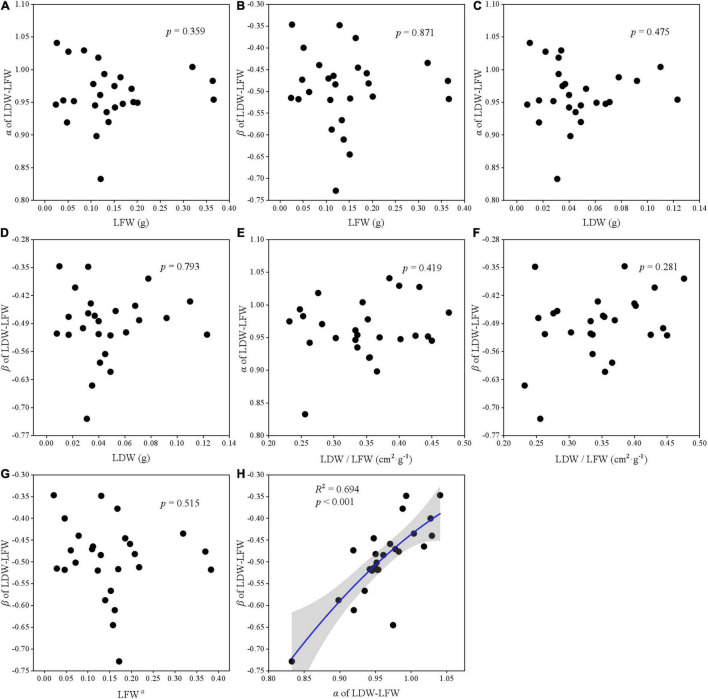
The correlation relationship of parameters of LDW and LFW. **(A)** The relationship between α and LFW; **(B)** the relationship between β and LFW; **(C)** the relationship between α and LDW; **(D)** the relationship between β and LDW; **(E)** the relationship between α and LDW/LFW; **(F)** the relationship between β and LDW/LFW; **(G)** the relationship between β and LFW*^α^*; and **(H)** the relationship between β and α.

**FIGURE 6:**
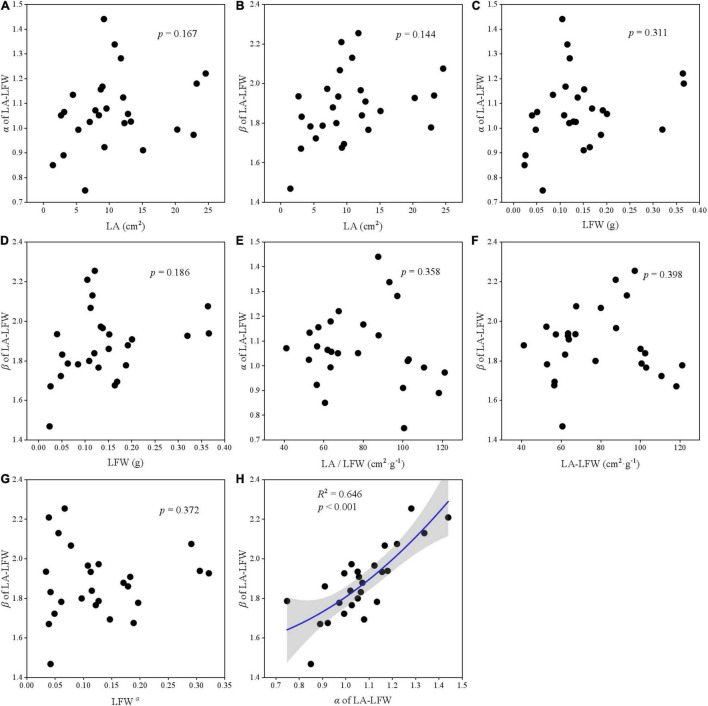
The correlation relationship of parameters of LA and LFW. **(A)** The relationship between α and LA; **(B)** the relationship between β and LA; **(C)** the relationship between α and LFW; **(D)** the relationship between β and LFW; **(E)** the relationship between α and LA/LFW; **(F)** the relationship between β and LA/LFW; **(G)** the relationship between β and LFW*^α^*; and **(H)** the relationship between β and α.

**FIGURE 7:**
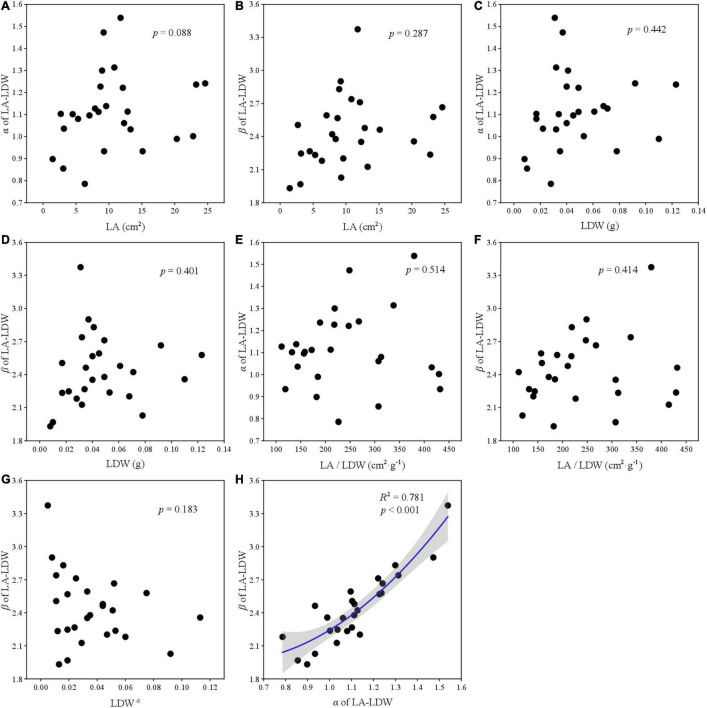
The correlation relationship of parameters of LA and LDW. **(A)** The relationship between α and LA; **(B)** the relationship between β and LA; **(C)** the relationship between α and LDW; **(D)** the relationship between β and LDW; **(E)** the relationship between α and LA/LDW; **(F)** the relationship between β and LA/LDW; **(G)**, the relationship between β and LDW*^α^*; and **(H)** the relationship between β and α.

The relationships among two scaling parameters (i.e., α and β) and other derived parameters were very complex (Figures 5–7). Overall, only the α and β of the three paired traits had a significant quadratic relationship (all *p* < 0.001) (Figures 5H, 6H, 7H), other traits and scaling parameters had no clear linear relationship (Figures 5A–G, 6A–G, 7B–G). Even so, we still detected a weakly relationship between α of LA-LDW with LA (*R*^2^ = 0.351, *p* = 0.088) (Figure 7A).

## Discussion

Plant traits response and adaptation to the environment are critical for plant survival. Combining with scaling relationship to explore the connection between leaf traits provides a theoretical basis for leaf variations with altitudinal gradient. Based on this study, we have found that plants adapt to altitude gradient mainly by adjusting LA and leaf mass, and the change of area and mass further affects scaling relationships.

### Effects of Altitude on Leaf Traits

Plants can produce adaptive strategies to cope with the environmental variation caused by the elevational gradient (Rudgers et al., 2019; Cui et al., 2020; Cubino et al., 2021). Previous studies had suggested that as elevation increases, the temperature always lowers, heat and energy supply limited LA expansion (Pan et al., 2013; Sun et al., 2017). But our study showed that the LA gradually increased up to 2,500 m. This non-intuitive pattern might be caused by the influence of precipitation on LA. The lowest elevation of our plots was located in a dry valley, where plants exhibited smaller leaves (Niinemets, 2001; Rudgers et al., 2019; Sun et al., 2020). As the altitude increased, precipitation and atmospheric humidity gradually increased, and LA also increased. Beyond a critical altitude (in our case, perhaps 2,500 m), temperature and heat may have limited LA increasing (Nikita et al., 2018). At low temperatures, smaller leaves reduce thermal convection in the boundary layer, which is very important for maintaining leaf heat and keeping the appropriate temperature of photosynthesis (Cubino et al., 2021; Lyu et al., 2021). LFW and LA have the same variation model. By comparison, LDW increased continuously with the altitude, and then gradually convergent. The increase of leaf biomass with altitude may reflect the conservative strategy of leaves, that is, the harsher the environment, the more investment in leaf biomass (Pan et al., 2013; Zhang et al., 2020; Yang et al., 2021). Abundant investment of leaf biomass promotes the denser mesophyll tissue, this not only helps prevent freezing injury but also reduces mechanical damage (Niinemets, 2001; Nikita et al., 2018).

In this study, SLA showed a unimodal change with altitude, the result is inconsistent with Umaña and Swenson (2019), who found that SLA of four out of six species decreased with elevation. This difference may cause by the relatively limited range of altitudes in their study (only ranged from 250 to 1,075 m a.s.l.). In addition, Costa et al. (2018) researched trait patterns along tropical elevation gradient ranging from 1,620 to 3,060 m also found that LA, SLA, and LDMC showed different patterns along their elevational gradient—sometimes decreasing, increasing, or showing no clear changes. Unimodal patterns of leaf traits may also reflect changes in biodiversity. Many studies have linked changes in leaf traits to species richness (Costa et al., 2018; Chen et al., 2021; Liu et al., 2021); competition among species may increase as species abundance increases. Furthermore, increased species abundance may promote niche differentiation, which can also influence the leaf traits pattern (Costa et al., 2018; Zhu et al., 2019; Guo et al., 2021). The biotas and succession along the altitudinal gradient may be one of the main factors leading to the variation of leaf traits.

For plants, leaf water content is associated with photosynthesis and light capture efficiency. Many studies have shown this for bamboos, climbing plants, and alpine plant taxa (Huang et al., 2019a,^2020^; Wang et al., 2021). The goodness of fit between LFW (*R*^2^ = 0.437) with altitude is greater than that LDW (*R*^2^ = 0.399) with altitude, which is consistent with the findings of other research. It may be due to the different leave shapes. Compared with broad leaves, narrow leaves require dense tissue (lower water content) to resist static loads. In other words, even given the same leaf fresh mass, different plants will have a great difference in leaf dry mass. Huang et al. (2019a) thought that the studies of leaf allometry had to consider the influence of foliar water content on the scaling relationship. However, in the field sampling process, it is difficult to obtain the leaves’ fresh weight in time, so there are still many operational difficulties.

### Effects of Altitude on α and β

Allometric relationships among leaf traits reflect their priority needs and dynamic growth. Based on the pooled data, the LDW did not keep pace with LFW, and as the leaf size gradually increased there was more biomass investment per unit area. The result of this study did not support the law of diminishing returns, which was inconsistent with Huang et al. (2019a,2020). This implied that with the increasing of LFW, leaf water content gradually increased or leaf dry matter content gradually decreased. It may be because larger LA transpiration more water and therefore need to store more water, and plenty of water keeps photosynthesis going. So, leaf water content and dry matter content will gradually increase, and the increased rate of water content is higher than that of dry matter content. Consistent with Yang et al.’s (2021) study, the fitness of LA–LFW is better than that of LA–LDW. Most plant—like evergreen and deciduous species have different hydraulic strategies and photosynthetic efficiency (Niinemets, 2001; Cubino et al., 2021; Wang et al., 2021), which may lead to differences in leaf water content or dry matter content, and thus lead to divergences in LDW per unit area.

To our knowledge, few studies reveal the variation of scaling parameters (i.e., scaling exponent and normalization constant) for leaf traits at such a large scale. Scaling relationships among leaf traits can reveal how the material allocation at leaf level as they grow. We found that most scaling exponents and normalization constants had no significant relationship between altitudes, except for the β of LA–LFW and LA–LDW. Our results were inconsistent with Pan et al. (2013) and Sun et al. (2017), who reported that scaling exponents for leaf mass and area significantly increased or was the V-shape with altitudinal gradient. These might be different vegetation types. Their study sites were located in subtropical monsoon regions, where most plants were evergreen. Previous studies indicate that evergreen and deciduous plants have different strategies to adapt to their habitat. The evergreen plants are resource-conserved and have greater leaf thickness and mass, lower SLA and water content, and longer leaf life span; and the deciduous are resource-acquisitive, with thinner leaves, greater SLA and lower dry matter content, and shorter leaf life (Li et al., 2008; Wu et al., 2013). Generally, evergreen plants have a higher biomass investment per unit area than deciduous plants (Lyu et al., 2021; Zhang et al., 2021). Another plausible explanation for this discrepancy may be because of the elevation range; the other studies included only 3 or 6 altitudes (Pan et al., 2013; Sun et al., 2017). If we had selected only a few elevations, we would found a significant linear relationship, too. In future studies, we suggest researchers consider elevation amplitude and study variation across more altitudes, vegetation types, and climatic regions.

Leaf mass and area are two important leaf traits for the most vascular plants. The relative changes of leaf mass and area reveal the metabolic activity and photosynthesis potential, which are not invariable (Liu et al., 2016; Umaña and Swenson, 2019; Sun et al., 2020). The scaling exponent and normalization constant for LDW–LFW, LA–LFW, and LA–LDW of this study were significant positive correlations. The altitude shifted leaf traits and affected α and β, it is not clear whether altitude, leaves, or their coupling relationship changes α and β. Along the altitudinal gradient, the environment changes rapidly over a short distance. Plants are subject to a lower temperature and higher irradiance and strong wind at higher altitudes (Nikita et al., 2018; Zhang et al., 2020; Kühn et al., 2021). However, in the middle altitude, most plants are understory, a few dominant species may be in the canopy. In other words, understory plants are rarely exposed to wind and strong light irradiance (Pan et al., 2013; Costa et al., 2018). Environmental variation at different stages of altitudinal gradient (high, middle, and low altitude) may be the main factors leading to the change of many traits that showed unimodal patterns.

### The Relationship Between α and β With Derived Parameters

Scaling relationships for leaves quantifies the allometry of resource allocation at the leaf scale and helps to interpret correlations among traits and scaling parameters (Thakur et al., 2019; Zhang et al., 2020). Our data showed close relationships between scaling exponent and normalization constant for all leaf traits. However, contrary to previous studies (Milla and Reich, 2007; Sun et al., 2017), LA did not affect α of LA–LFW and LA–LDW, LFW also did not affect α of LDW–LFW. The reason might be that our target plant species included conifer species such as *Abies fabri*, *Pinus bungeana*, and *Cupressus chengiana*, and our results were the pooled data rather than life-form or plant-specific.

In our study, we included conifer species, whose unusual leaf morphology may have influenced the results of our analysis. For example, they have very small LA (needle or scale leaf), specific LA, and water content, but have large leaf mass. They may be a very strong disturbance when compared with broad-leaved species, and we recommend that future studies treat broad-leaved and coniferous species separately.

## Conclusion

With the increase of altitude, the LFW and LA showed a unimodal change, while the LDW was a slow increase. LA with LFW and LDW showed an increasing returns relationship. Our study demonstrates that there is no fixed variation pattern of scaling relationship with altitude, and leaf traits had little effect on the variation of scaling relationship. Therefore, the coordination of leaf trait variation and scaling relationship may be the positive response of plants to the elevation gradient. These provide a possible biological explanation for the plant adaptation to high radiation, freezing, and strong wind on altitude gradient.

## Data Availability Statement

The original contributions presented in the study are included in the article/Supplementary Material, further inquiries can be directed to the corresponding author.

## Author Contributions

KY, GC, and WC designed the experiments, were responsible for field collections, and analyzed the data. KY, GC, JX, and WC wrote and revised the manuscript. All authors contributed to the article and approved the submitted version.

## Conflict of Interest

The authors declare that the research was conducted in the absence of any commercial or financial relationships that could be construed as a potential conflict of interest.

## Publisher’s Note

All claims expressed in this article are solely those of the authors and do not necessarily represent those of their affiliated organizations, or those of the publisher, the editors and the reviewers. Any product that may be evaluated in this article, or claim that may be made by its manufacturer, is not guaranteed or endorsed by the publisher.

## Footnotes

1
http://www.iplant.cn/frps

2
https://imagej.net/software/fiji/
